# A Bayesian framework for health economic evaluation in studies with missing data

**DOI:** 10.1002/hec.3793

**Published:** 2018-07-03

**Authors:** Alexina J. Mason, Manuel Gomes, Richard Grieve, James R. Carpenter

**Affiliations:** ^1^ Department of Health Services Research and Policy London School of Hygiene and Tropical Medicine London UK; ^2^ Department of Medical Statistics London School of Hygiene and Tropical Medicine London UK; ^3^ MRC Clinical Trials Unit University College London London UK

**Keywords:** Bayesian analysis, cost‐effectiveness analysis, expert elicitation, missing not at random, pattern‐mixture model

## Abstract

Health economics studies with missing data are increasingly using approaches such as multiple imputation that assume that the data are “missing at random.” This assumption is often questionable, as—even given the observed data—the probability that data are missing may reflect the true, unobserved outcomes, such as the patients' true health status. In these cases, methodological guidelines recommend sensitivity analyses to recognise data may be “missing not at random” (MNAR), and call for the development of practical, accessible approaches for exploring the robustness of conclusions to MNAR assumptions.

Little attention has been paid to the problem that data may be MNAR in health economics in general and in cost‐effectiveness analyses (CEA) in particular. In this paper, we propose a Bayesian framework for CEA where outcome or cost data are missing. Our framework includes a practical, accessible approach to sensitivity analysis that allows the analyst to draw on expert opinion.

We illustrate the framework in a CEA comparing an endovascular strategy with open repair for patients with ruptured abdominal aortic aneurysm, and provide software tools to implement this approach.

## 1 INTRODUCTION

Health economic evaluations use evidence from multiple sources including meta‐analyses, single randomised controlled trials (RCTs), and observational studies. A general concern is missing data, for example, because patients are lost to follow‐up or fail to provide complete responses to questions about their health status or resource use. Multiple Imputation has been widely recommended for handling missing data (Briggs, Clark, Wolstenholme, & Clarke, 2003; Faria, Gomes, Epstein, & White, 2014; Gomes, Diaz‐Ordaz, Grieve, & Kenward, 2013; Hunter et al., 2015; Hughes et al., 2016), but typically assumes that data are “missing at random” (MAR). 1Although we adopt standard terminology such as “data are MAR,” by this we mean that the mechanism that we are willing to assume for how the missing values arise is MAR.


Broadly speaking, the MAR assumption can be expressed in two ways (Carpenter & Kenward, 2013, Ch. 1). First, the probability that data (e.g., on health outcomes) are observed is independent of their true value, conditional on the fully observed data (selection perspective). A second perspective and one we use in this paper is that given the fully observed data, the distribution of potentially missing data does not depend on whether those data are observed (pattern‐mixture perspective).

Unfortunately, the MAR assumption cannot be verified from the data collected within the study. Therefore, it is important to assess the robustness of conclusions to plausible departures from MAR—in other words to plausible Missing Not At Random (MNAR) mechanisms. Thus, methodological guidelines for handling missing data recommend that studies undertake sensitivity analyses to recognise that the data may be MNAR (Little et al., 2012; Sterne et al., 2009), and call for the development of relevant, accessible methods for such sensitivity analysis.

This is particularly relevant for studies that report self‐reported outcomes, such as health‐related quality of life (QoL; Faria et al., 2014; Fielding, Fayers, McDonald, McPherson, & Campbell, 2008), where the chance of observing the patient's QoL and their true QoL are both likely to be driven by their health status at that time, which is unobserved. However, although there have been numerous papers in the general econometrics and biostatistics literatures proposing MNAR models, recent reviews of published economic evaluations found that these methods are rarely adopted (Gabrio, Mason, & Baio, 2017; Leurent, Gomes, & Carpenter, 2018; Noble, Hollingworth, & Tilling, 2012). This limitation in the way missing data are handled in cost‐effectiveness analyses (CEA) extends to other empirical health economics studies that rely on information collected from patients and the public, such as comparisons of provider performance (Gomes, Gutacker, Bojke, & Street, 2016), evaluations of health policies (Grieve, Sekhon, Hu, & Bloom, 2008), or studies that elicit patient preferences (Ryan, Gerard, & Amaya‐Amaya, 2008).

This paper aims to address this issue by proposing and illustrating a practical Bayesian framework for the analysis of CEA data with a nontrivial proportion of missing values. Our framework makes use of our recently developed web‐based tool for eliciting information about missing values from experts (Mason et al., 2017), and we further provide annotated WinBUGS code to allow others to implement the approach.

An advantage of undertaking the analysis from a Bayesian perspective is it naturally allows uncertainty about the missing data mechanism to be propagated through into the eventual measures of decision uncertainty. In particular, a fully Bayesian MNAR analyses can directly incorporate expert opinion about differences between those with missing versus observed outcome data (Garthwaite, Kadane, & O'Hagan, 2005; O'Hagan et al., 2006) through specifying prior distributions; see, for example, White, Carpenter, Evans, and Schroter (2007) and Smuk, Carpenter, and Morris (2017). Although fully Bayesian methods for handling MNAR have not been previously considered in health economics studies, this approach builds on Bayesian approaches to CEA more generally (Baio, 2014; Grieve, Nixon, & Thompson, 2010; Nixon & Thompson, 2005; O'Hagan & Stevens, 2001; Thompson & Nixon, 2005). In particular, Lambert, Billingham, Cooper, Sutton, and Abrams (2008) discussed Bayesian models for dealing with incomplete costs, but assumed the data were MAR.

A common barrier to the use of fully Bayesian approaches is the lack of practical tools for eliciting expert opinion (Cooper, Spiegelhalter, Bujkiewicz, Dequen, & Sutton, 2013; Hadorn, Kvizhinadze, Collinson, & Blakely, 2014). Elicitation methods previously developed for single parameters in decision models (Leal, Wordsworth, Legood, & Blair, 2007) are not applicable to settings, such as CEA, that use patient‐level data. In such settings, it is necessary to recognise that parameters may differ by treatment groups, and may be correlated with one another. Previous elicitation studies have used group consensus, for example, Delphi methods, but these are relatively time consuming and difficult to implement (Sullivan & Payne, 2011). However, we have recently addressed this by developing a practical web‐based tool for eliciting the expert opinion required for CEA that use RCT data (Mason et al., 2017).

We now build on this to present a practical Bayesian framework for the analysis of data from a health economics study with missing data. We illustrate this framework in the analysis of CEA data from a multicenter RCT (IMPROVE) that compared an emergency endovascular strategy (eEVAR) with open repair for patients with ruptured abdominal aortic aneurysm (Grieve et al., 2015). This study found that the eEVAR strategy was cost‐effective, due to improvements in average QoL, but these data were missing for 20% (3 months follow‐up) and 24% of patients (12 months). Although Grieve et al. (2015) used Multiple Imputation, assuming values were MAR, it is unclear whether the conclusion that eEVAR is relatively cost‐effective is sensitive to alternative assumptions about the missing data. Hence, this study exemplifies the more general requirement for a framework for addressing missing data in CEA.

The remainder of the paper proceeds as follows. Section 2 describes the strategy for the sensitivity analysis, illustrated using the IMPROVE study in Section 3. Section 4 discusses the findings and provides some suggestions for further research.

## 2 FRAMEWORK FOR SENSITIVITY ANALYSIS

**Figure 1:**
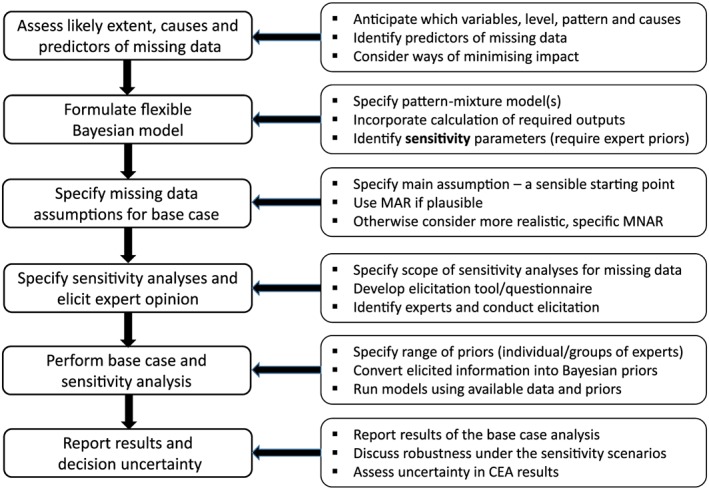
Key steps in our proposed sensitivity analysis framework for addressing data that are MNAR in cost‐effectiveness analysis. CEA: cost‐effectiveness analyses; MAR: missing at random; MNAR: missing not at random [Colour figure can be viewed at http://wileyonlinelibrary.com]

Our proposed framework follows general methodological recommendations (Little et al., 2012; White, Horton, Carpenter, & Pocock, 2011) tailored to the particular requirements of CEA that use data from a RCT. Figure 1 summarises the key steps of our proposed strategy. Briefly:

1. At the design stage, use previous studies and expert opinion to anticipate the extent, pattern, and causes of missing data. Identify predictors of the missing data and incorporate into the data collection, and minimise the potential for missing data.
2. Formulate the statistical model for the analysis.
3. In the protocol, specify a contextually plausible assumption about the missing data for the base case analysis.
4. Specify the scope of sensitivity analyses for the missing data, and collect prior information from experts to inform these.
5. When the data are available, perform the base case and sensitivity analysis.
6. Report the results of the base case analysis, and discuss the extent to which they are robust under the sensitivity scenarios.

### 2.1 A flexible statistical model to facilitate sensitivity analysis

We extend Bayesian approaches for CEA that use RCTs to settings with missing data (Baio, 2013; Nixon & Thompson, 2005). At baseline, and at scheduled times over the course of the trial follow‐up, we observe QoL_*ij*_, from patient *i* = 1,…,*n* at follow‐up visit *j* = 1,…, *J*, where patient *i* is randomised to intervention arm *k* = 1,…, *K*, where *k* = 1 is the reference, or control, arm. We also observe a vector of baseline covariates **x**_*i*_ for each patient.

We assume that the cost is the aggregate cost calculated over the requisite time horizon, and denote this by Cost_*i*_. To construct the quality‐adjusted life year (QALY), a generic outcome measure typically required by decision makers, QoL measurements are combined with patient's survival. This weighted process is often described as

(1) $${\text{QALY}}_{i}=\sum_{j=1}^{J}{\text{QoL}}_{ij}\times t_{ij}$$

where *t*_*ij*_ is the duration of the *j*th follow‐up assessment expressed as a fraction of 1 year (Manca, Hawkins, & Sculpher, 2005).

The general statistical model has two parts: (a) a multivariate model for the repeated QoL scores measured on the individual patients over time and (b) a bivariate model of costs and effects, allowing the calculation of treatment effects. For ease of exposition, the first part is specified as an arm‐specific unstructured multivariate normal model, but the framework allows for alternative distributions (e.g., Basu & Manca, 2012, propose using a Beta distribution).

(2) $$\begin{array}{ccc} \left(\begin{array}{c} {\text{QoL}}_{i1}\\ {\text{QoL}}_{i2}\\ \vdots \\ {\text{QoL}}_{iJ} \end{array}\right) & ∼MVN\left[\left(\begin{array}{c} \mu_{i1}\\ \mu_{i2}\\ \vdots \\ \mu_{iJ} \end{array}\right),\Sigma_{k}\right] & \\ \mu_{ij} & =x_{i}^{T}\beta_{jk}. \end{array}$$

For the second part of the model, we have

(3) $$\begin{array}{ccc} \left(\begin{array}{c} {\text{QALY}}_{i}\\ {\text{Cost}}_{i} \end{array}\right) & ∼BVN\left[\left(\begin{array}{c} \nu_{i}^{q}\\ \nu_{i}^{c} \end{array}\right),\Omega \right] & \\ \nu_{i}^{q} & =\gamma_{k}^{q}\\ \nu_{i}^{c} & =\gamma_{k}^{c} \end{array}$$

where *k* = 1,…,*K* indexes the intervention arm. Treatment contrasts can be estimated as differences between the ***γ*** parameters.

Model (3) is our substantive model, which may be extended to include covariates, from which inferences for quantities such as the incremental net benefit can be directly derived. Model (2) is essentially an imputation model for missing QoL values. The covariates are included to reduce the variability of the imputed data and increase the plausibility of the MAR assumption. For the fully Bayesian approach adopted in our framework, both models are fitted simultaneously by linking *ν* ^*q*^ and Ω in (3) to ***μ***_*j*_ and Σ in (2). An example of this is provided in the discussion of the statistical model for our application in Section 3.

We can choose vague priors for the regression parameters, and in general a Wishart prior with minimum degrees of freedom for Σ_*k*_. For bivariate data, we show in Appendix A how to specify (3) using a conditional step; this allows us more flexibility over the prior for the elements of Ω, and facilitates extensions to gamma models for costs.

### 2.2 Sensitivity analysis model

Methodological guidelines suggest three broad approaches to sensitivity analysis for missing data, that is, exploring the robustness of inferences to the possibility that the distribution of the missing values may differ from that of the observed ones. These are latent variable models, selection models, and pattern‐mixture models (Molenberghs et al., 2015, Ch. 15). Here, we focus on the pattern‐mixture approach, because this approach —which looks at each pattern of missing data in turn—allows us to make relevant, accessible assumptions to explore plausible departures from MAR.

For our general sensitivity analysis model, we modify *μ*_*ij*_ and $\nu_{i}^{c}$ from Equations 2 and 3 as follows:

(4) $$\begin{array}{ccc} \mu_{ij} & =x_{i}^{T}\beta_{jk}+(1-R_{ij})\Delta_{jk} & \\ \nu_{i}^{c} & =\gamma_{k}^{c}+(1-S_{i})\Gamma \end{array}$$

where *R*_*ij*_ = 1 if QoL_*ij*_ is observed and 0 otherwise; *S*_*i*_ = 1 if patient *i*'s cost is observed and 0 otherwise, and *k* as before.

Writing **Δ**_*k*_ = (Δ_1*k*_,…,Δ_*Jk*_)^*T*^, and $\Lambda ={\left(\Delta_{1}^{T},\text{⋯},\Delta_{K}^{T},\Gamma \right)}^{T},$ we can assume the sensitivity parameters are distributed

(5) $$\Lambda ∼MVN(\mu_{\Lambda },\Sigma_{\Lambda })$$

Each of the Δ_*ijk*_ parameters represents the average (marginal) difference between the observed and missing values of QoL_*ijk*_. However, the distribution implied for any individual missing value will also be influenced by the observed QoLs for that individual, through the covariance matrix Σ_*k*_.

### 2.3 Choosing sensitivity parameters

The number of sensitivity parameters can quickly become large with increasing time‐points. Because none of these parameters are observed, a sensible strategy is to discuss with subject‐matter experts which of the parameters are most relevant, and how to plausibly inform the choice of values. For example, researchers may decide to focus on sensitivity parameters early in the follow‐up, where clinical experts have more frequent interaction with patients and are more likely to recall possible (unmeasured) reasons for the missing data. Also, if the intervention is for an acute illness, the researchers may anticipate that the results will be relatively sensitive to any change in short term outcomes, whereas for a chronic illness, longer term outcome may be of greater importance. In addition, one may wish to focus on specific outcomes that are more likely to be MNAR, such as QoL, and assume MAR for others such as costs.

Our approach for determining the choice of values for the sensitivity parameters is to elicit information about the Λ's from experts. A particular strength of this approach is that it allows us to quantify how experts would interpret the study, taking into account their (often implicit) understanding of the differences between the observed and missing QoL values.

There are alternatives (Carpenter, Roger, & Kenward, 2013; Carpenter & Kenward, 2013, Ch. 10), and here we briefly describe two. The first, often known as the “delta” method (Faria et al., 2014; Leurent, Gomes, Faria, et al., 2018), sets **Σ**_Δ_ = 0 and typically all the Δ_*jk*_ to be equal. Sensitivity analysis then proceeds by increasing Δ until a “tipping point”, Δ_tip_, is reached (e.g., the intervention is no longer cost‐effective) and then discussing the plausibility of Δ_tip_. Variants of this include only the first of a patient's missing values to be changed by Δ, or changing the first missing value by Δ, the second by 2Δ, and so on, thus reflecting the time of dropout. Although this approach can be justified in some settings, a general criticism is that it is implausible that **Σ**_Δ_ = 0. A second approach is to specify the Δ's implicitly, by reference to other arms or groups of patients within the study. For instance, active patients with missing values can be imputed as if they belong to the reference arm. One limitation of the reference‐based approach is that it is not very intuitive/straightforward for intermittent missing data, because it is more challenging to make plausible references to other treatment arms intermittently over time. Our approach has the potential to make more plausible assumptions about the missing data compared with the delta method and the reference‐based approach, because it provides the flexibility to incorporate external/additional information (e.g., expert opinion) about the reasons for the missing data.

A “strategy” to simplify the number of sensitivity parameters has been already outlined above. In the simplest setting, focusing on one outcome (QoL), a two‐arm trial and one time point (J=1), there will be five parameters to elicit: the two components, *μ*_1_,*μ*_2_ of ***μ***_Λ_, and the three components (*σ*_1_,*σ*_2_,*ρ*) of **Σ**_Λ_.

In practice, further challenges arise eliciting information for these parameters for *J*>1 time points. An alternative is to use the information elicited about Δ at a single time‐point to inform the sensitivity parameters at other follow‐up assessments, for example, by drawing from the same distribution or using (possibly varying) multiples of Δ.

### 2.4 Elicitation

Eliciting expert opinion involves (a) identifying experts whose knowledge about the prognosis and outcomes of the patients in the RCT with missing data exceeds the information in the available data, (b) developing a suitable approach for eliciting expert opinion, and (c) conducting the elicitation and synthesising the responses.

With individual elicitation, each expert gives their view independently, and these views are expressed numerically and then appropriately aggregated. For sensitivity analysis, this approach is desirable because (a) the purpose is to elicit probability distributions for sensitivity parameters, rather than agreeing on a single value, and (b) it encourages more experts to participate; see O'Hagan et al. (2006) and White (2015) for further details. The alternative, group elicitation, allows experts to debate and reach consensus about the elicited values, but is more time consuming and limits the number and range of experts included.

Using individual elicitation, sensitivity analysis can be based on different priors, including distinct individual priors to retain diversity of opinion and one or more pooled priors that combine the opinion of multiple experts. Our mathematical aggregation method is linear pooling (O'Hagan et al., 2006).

To convert expert opinion about the differences between patients with observed and missing outcomes into an informative prior, we must elicit an expected value or point estimate, which corresponds to the value that the expert believes is most likely, together with the expert's uncertainty about this estimate. In the context of a CEA that uses RCT data, estimates are required by randomised arm, together with an estimate of the correlation between treatment arms. We have recently developed, piloted, and made available a web application to do this, which successfully elicited prior distributions for *μ*_1_,*μ*_2_ and the three components *σ*_1_,*σ*_2_,*ρ* (Mason et al., 2017).

## 3 APPLICATION TO THE IMPROVE STUDY

Using the IMPROVE study, we now illustrate how to use our elicitation tool and implement the proposed sensitivity analysis framework using a fully Bayesian approach.

### 3.1 Overview

IMPROVE was a multicenter RCT and CEA that investigated whether eEVAR was effective and cost‐effective versus open repair for patients with suspected ruptured aortic aneurisms (see Grieve et al., 2015 for details). The published CEA reported that eEVAR led to an increase in average QoL (EQ‐5D‐3L) at both 3 and 12 months post randomisation, and a reduction in average costs up to 12 months, leading to a positive incremental net benefit (INB) of £3877 (95% CI: £253, £7,408) assuming a willingness‐to‐pay of 30,000GBP per QALY gain.

However, the IMPROVE trial illustrates a number of typical missing data issues in CEA. Although all deaths are recorded, of the 301 eligible patients who survived to 12 months, 20% and 24% of patients failed to return their QoL questionnaires at 3 and 12 months, respectively. In addition, some patients were missing data for costs (over 40%) and for a baseline prognostic covariate, the Hardman index (a disease‐specific morbidity score; over 10%). Overall, of the eligible patients who survived to 12 months, only 138 (46%) had complete data for costs, QoL, and the Hardman index.

### 3.2 Step 1: Anticipate and reduce the extent of missing data at the design stage

At the design stage of the IMPROVE trial, missing data were anticipated for the QALY and cost endpoints because clinical trials often fail to collect all intended information about QoL and resource use variables. Regarding the costs, data on the main resource use components such as hospital length‐of‐stay and medical devices were planned to be collected using case‐report forms. In this context, costs were anticipated to be Missing Completely At Random because any missing data were expected to be due to administrative errors unrelated to patients' incurred costs. Questionnaires for community care use were expected to lead to a higher proportion of missing costs, but these community costs were a very small component of the total cost.

On the other hand, QoL measures were reliant on patient‐reported questionnaires administered at 3 and 12 months follow‐up, and hence we anticipated a higher proportion of missing data. In discussion with clinical experts and trial coordinators, it was recognised that the MAR assumption would be less plausible for the QoL endpoint, because the chance of completing the EQ‐5D‐3L questionnaires might be related to the patient's true health status. However, there was little evidence about a clear MNAR mechanism in this context (literature and/or expert opinion). As a result, we carefully considered the collection of important patient characteristics, such as age and Hardman Index that were anticipated to be strong predictors of both QoL and the probability of observing the data. By measuring and including such variables in the analysis, this would help make the MAR assumption more plausible for the base case analysis.

In addition, we have taken a number of active steps to reduce the extent of missing data. For example, we have encouraged patients to complete their resource use and QoL questionnaires during follow‐up appointments and provided assistance when requested. In addition, patients were sent reminders (phone calls) whenever they have failed to return their completed questionnaires.

### 3.3 Step 2: Statistical model

We illustrate our approach with the 12 month survivors from the IMPROVE trial, eligible for follow‐up. For these patients, QALY is calculated as a combination of two QoL scores (at 3 and 12 months), QoL_*i*1_ and QoL_*i*2_. Therefore, including covariates, the base case analysis models for the QoL (2) and cost‐effectiveness endpoints (3) are refined as

(6) $$\begin{array}{ccc} \left(\begin{array}{c} {\text{QoL}}_{i1}\\ {\text{QoL}}_{i2} \end{array}\right) & ∼BVN\left[\left(\begin{array}{c} \mu_{i1}\\ \mu_{i2} \end{array}\right),\left(\begin{array}{cc} \sigma_{1}^{2} & \varrho \sigma_{1}\sigma_{2}\\ \varrho \sigma_{1}\sigma_{2} & \sigma_{2}^{2} \end{array}\right)\right] & \\ \mu_{i1} & =\eta_{1k}+\beta_{a1}age_{i}+\beta_{s1}sex_{i}+\beta_{h1,hardman(i)}\\ \mu_{i2} & =\eta_{2k}+\beta_{a2}age_{i}+\beta_{s2}sex_{i}+\beta_{h2,hardman(i)}\\ \left(\begin{array}{c} {\text{QALY}}_{i}\\ {\text{Cost}}_{i} \end{array}\right) & ∼BVN\left[\left(\begin{array}{c} \nu_{i}^{q}\\ \nu_{i}^{c} \end{array}\right),\left(\begin{array}{cc} \sigma_{q}^{2} & \rho \sigma_{q}\sigma_{c}\\ \rho \sigma_{q}\sigma_{c} & \sigma_{c}^{2} \end{array}\right)\right]\\ \nu_{i}^{q}=f(\mu_{i1},\mu_{i2}) & =\frac{1}{2}\mu_{i1}+\frac{3}{8}\mu_{i2}\\ \sigma_{q}=f(\sigma_{1},\sigma_{2},\varrho ) & =\frac{1}{4}\sigma_{1}^{2}+\frac{3}{8}\varrho \sigma_{1}\sigma_{2}+\frac{9}{64}\sigma_{2}^{2}\\ \nu_{i}^{c} & =\eta_{3k}+\beta_{a3}age_{i}+\beta_{s3}sex_{i}+\beta_{h3,hardman(i)} \end{array}$$

where ***η*** are treatment specific intercepts, *k* = 1,2 indexes the eEVAR and open repair groups, and *h* *a* *r* *d* *m* *a* *n*(*i*) = 1,…,5 is the Hardman index indicator for individual *i*. All other parameters, including *σ*_*c*_, are given vague priors.

To enable a fully Bayesian approach, the joint models for the QoL imputation and substantive models are directly linked through the parameters for the QALY distributions. Three covariates are included in both models: *a* *g* *e* and *s* *e* *x* are fully observed, but *h* *a* *r* *d* *m* *a* *n* is incomplete, requiring the addition of a covariate imputation model (see Supporting Information for details).

This model is fitted using the WinBUGS software (Lunn, Thomas, Best, & Spiegelhalter, 2000), and for ease of implementation is specified using a conditional formulation for the bivariate data. Details are provided in Appendix A, including prior specification.

### 3.4 Step 3: Missing data assumption for the base case analysis

Drawing on the protocol for the CEA analysis, discussed in Step 1, the base case analysis assumed both costs and QoL were MAR. We started by investigating missing data patterns and observed predictors of missing data across both cost‐effectiveness endpoints, in order to better understand the actual reasons for the missing data and the need to assess possible departures from the MAR assumption. Table 1 reports the overall proportion of missing data in the 1‐year cost‐effectiveness endpoints, which is slightly higher for the open repair arm. As typical in CEA, the IMPROVE study reported an intermittent missing data pattern (Table S1), for example, with some patients having missing QoL at 3‐months but not at 12‐months, and vice‐versa.

**Table 1:** Level of missing data by treatment arm for eligible patients who survived to 12 months in the IMPROVE trial

	eEVAR	Open repair	Total
Number of patients	161	140	301
Outcomes: *n* (%) missing	
EQ‐5D at 3 months	27 (17)	33 (24)	60 (20)
EQ‐5D at 12 months	34 (21)	38 (27)	72 (24)
Costs at 12 months	72 (45)	69 (49)	141 (47)
Baseline covariates: *n* (%) missing	
Hardman Index	16 (10)	20 (14)	36 (12)

*Note*. Age, sex, and time to death are also used in the cost‐effectiveness analyses, but are excluded from this table as they are fully observed where required. eEVAR: endovascular strategy.

There was evidence from an analysis of the IMPROVE data that the missing data predictors differed between cost‐effectiveness endpoints and over time. For example, there was some evidence that the chance of observing QoL at 3 months was related to patients' hospital length‐of‐stay, whereas at 12 months, other patient characteristics, such as age and blood pressure, were also predictive of missingness. As anticipated at the design stage, the probability of observing costs seemed to be unrelated to patients' baseline characteristics.

### 3.5 Step 4: Sensitivity analysis

We allow the QoL endpoint to be MNAR and modify the statistical model in (6) to include treatment specific sensitivity parameters in the specification of *μ*_*i*1_ and *μ*_*i*2_ as described in Section 2.2, with the simplifying assumption that the Δ are time invariant. To inform the priors for these sensitivity parameters, for the IMPROVE trial we designed a questionnaire to elicit opinion from clinical experts. A full description of the elicitation is reported elsewhere (Mason et al., 2017). Briefly, the main purpose of the elicitation was to quantify differences in the mean QoL score between patients who did, and did not, complete QoL questionnaires at 3 months, across the eEVAR and open repair arms. To allow for the possibility that the elicited values in the two arms are correlated, for example, expert belief in a high QoL score for the open repair arm may make a high score in the eEVAR arm more likely, we also asked experts about the score in the eEVAR arm conditional on a given score for the open repair arm. The questionnaire followed general recommendations for the design of elicitation surveys (Johnson, Tomlinson, Hawker, Granton, & Feldman, 2010; O'Hagan et al., 2006).

We developed a web‐based elicitation tool, in which the questionnaire was embedded, using a web application framework (Shiny package) within the widely used statistical software, R (Chang, Cheng, Allaire, Xie, & McPherson, 2015; RStudio, Inc, 2015). We framed the main questions in terms of “typical” IMPROVE patients and asked the expert for their most likely QoL and to indicate graphically their uncertainty about this level. We represented the distribution of their views according to a normal distribution. We invited 46 experts, comprising trial investigators and coordinators (including surgeons and vascular nurses) from the IMPROVE trial to complete the questionnaire either online or face‐to‐face at The Vascular Society Annual Scientific Meeting (2015).

**Figure 2:**
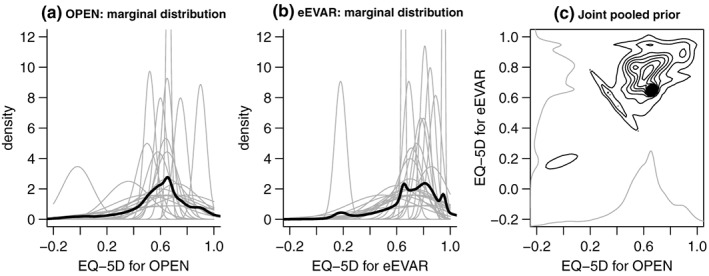
Individual and pooled prior distributions for patients randomised to endovascular strategy (eEVAR) and open repair (OPEN) treatment groups. In (a) and (b), thin grey lines: individual priors; thick black lines: pooled priors across all experts. In (c), the black contour lines show the joint pooled prior, and the grey lines indicate the underlying marginal distributions. Although each individual prior has been elicited as a normal distribution, this restriction does not apply to the pooled priors that are a mixture of normal distributions

Twenty‐six experts completed the survey, 15 face‐to‐face, 11 online, of whom 17 were doctors and 9 nurses. Figure 2a,b shows the range of expert belief about plausible values for patients with missing QoL data randomised to the eEVAR and open repair treatment arms respectively. The grey lines represent the views of individual experts, and the thick black lines their combined views. Figure 2c shows the pooled prior based on all experts as a series of contours, with the underlying marginal distributions superimposed on the axes. This pooled prior is an average of the individual distributions, based on linear pooling with equal weights, and specified as a mixture of the bivariate normal distributions for each expert.

We also formulated pooled priors according to the expert's background (doctors vs. nurses), and mode of completion (face‐to‐face vs. online). We explored the sensitivity of the results to individual priors, namely, the most sceptical and enthusiastic experts (believed QoL score 0.2 higher for open repair and 0.29 higher for eEVAR, respectively), the most and least certain experts.

### 3.6 Step 5: Analysis

All the models were run with two chains initialised using diffuse starting values to produce a sample of 50,000 after convergence for posterior inference. Convergence is assumed if the Gelman–Rubin convergence statistic (Brooks & Gelman, 1998) for individual parameters is less than 1.05 and a visual inspection of the trace plot for each parameter is satisfactory.

Table 2 reports the results of the sensitivity analysis, MAR, and complete case analysis. The incremental QALYs and costs include decedents and patients ineligible to follow‐up, as well as 12‐month survivors (see Supporting Information for model details).

**Table 2:** Differences between randomised arm in the mean QoL at 3 months, mean QoL at 12 months, mean QALYs and mean costs (eEVAR ‐ open repair)

		INC QoL 3 months^a^			INC QoL 12 months^b^			INC QALY^c^			INC COST^d^ (GBP)	
Number of Patients			318			301			613			613
Complete Case Analysis (CCA)		0.079	(−0.004, 0.162)		0.069	(−0.012, 0.152)		0.057	(0.035, 0.080)		−1147	(−3519, 1216)
Base case analysis (MAR)		0.063	(−0.005, 0.130)		0.046	(−0.021, 0.113)		0.043	(0.018, 0.067)		−704	(−3189, 1766)
Sensitivity analysis (MNAR)												
all experts (25 experts)		0.072	(−0.058, 0.199)		0.061	(−0.085, 0.202)		0.049	(−0.009, 0.103)		−689	(−3137, 1790)
doctors (17 experts)		0.082	(−0.037, 0.194)		0.071	(−0.060, 0.196)		0.053	(0.001, 0.101)		−695	(−3158, 1793)
nurses (8 experts)		0.056	(−0.099, 0.224)		0.043	(−0.134, 0.234)		0.041	(−0.028, 0.117)		−699	(−3160, 1773)
face‐to‐face delivery (15 experts)		0.075	(−0.080, 0.222)		0.064	(−0.111, 0.231)		0.050	(−0.019, 0.115)		−702	(−3179, 1781)
online delivery (10 experts)		0.073	(−0.013, 0.172)		0.062	(−0.030, 0.170)		0.049	(0.013, 0.091)		−704	(−3177, 1765)
Extreme sensitivity analysis (MNAR)												
sceptical expert		0.005	(−0.153, 0.160)		−0.016	(−0.194, 0.160)		0.018	(−0.052, 0.087)		−701	(−3166, 1770)
enthusiastic expert		0.111	(0.052, 0.169)		0.106	(0.047, 0.164)		0.066	(0.044, 0.088)		−696	(−3160, 1772)
most certain expert		0.049	(−0.006, 0.103)		0.034	(−0.019, 0.087)		0.038	(0.018, 0.058)		−694	(−3158, 1781)
least certain expert		0.069	(−0.059, 0.196)		0.058	(−0.083, 0.199)		0.047	(−0.007, 0.102)		−703	(−3179, 1745)

*Note*. Comparison of models and priors, posterior mean (95% credible interval) shown. eEVAR: endovascular strategy; MAR: missing at random; MNAR: missing not at random; QALY: quality‐adjusted life year; QoL: quality of life.

a INC QoL 3mths: incremental QoL at 3 months from open repair arm to eEVAR arm.

b INC QoL 12mths: incremental QoL at 12 months from open repair arm to eEVAR arm.

c INC QALY: incremental QALY from open repair arm to eEVAR arm.

d INC COST: incremental costs from open repair arm to eEVAR arm.

### 3.7 Step 6: Interpretation of results

Most MNAR scenarios reported a somewhat higher incremental QoL at both 3 and 12 months, and wider 95% credible intervals, compared with the MAR scenario. The combined opinion from nurses led to a smaller incremental QoL than that of doctors. Experts elicited at conference and online produced similar results. The MAR and MNAR scenarios provided similar incremental QALY, but the latter provided greater uncertainty. The different assumptions about the missing QoL data led to similar incremental costs across scenarios.

**Figure 3:**
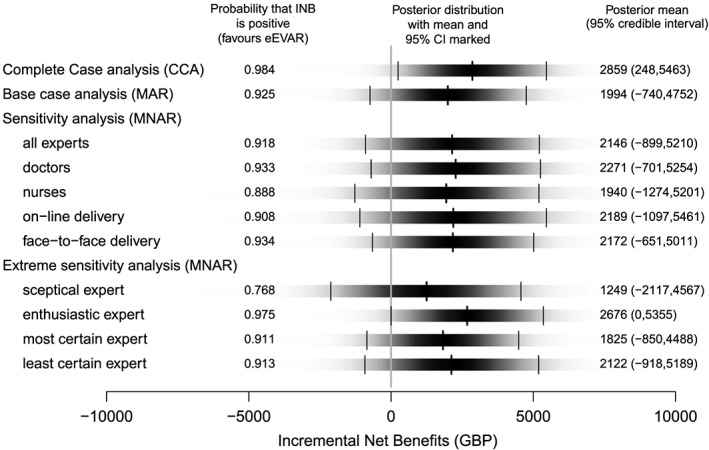
INBs across different departures from MAR. Each shaded rectangular strip shows the full posterior distribution of the incremental net benefits, valuing quality‐adjusted life year gains at 30,000GBP per quality‐adjusted life year. The darkness at a point is proportional to the probability density, such that the strip is darkest at the maximum density and fades into the background at the minimum density. The posterior mean and 95% credible interval are marked. eEVAR: endovascular strategy; INBs: incremental net benefits; MAR: missing at random; MNAR: missing not at random [Colour figure can be viewed at http://wileyonlinelibrary.com]

We also report the INB (assuming a willingness‐to‐pay of 30,000GBP per QALY gain) and the probability of eEVAR being cost‐effective, across the different scenarios. Figure 3 shows the full posterior distribution of INB as a density strip, with the mean and 95% credible interval marked (Jackson, 2008). The probability that eEVAR is cost‐effective (i.e., the INB is positive) remains above 0.75 across the alternative departures from MAR, including the scenario where the priors are based on the most sceptical expert.

## 4 DISCUSSION

This paper proposes and illustrates a flexible Bayesian framework for conducting sensitivity analysis to potential departures from MAR, allowing more realistic assumptions to be made about missing data in health economic evaluation. As part of this framework, we develop a fully Bayesian pattern‐mixture model to allow for potential departures from MAR. We provide a strategy, and accompanying web‐based tool, to elicit expert opinion and use this to define the requisite priors within the Bayesian model. This sensitivity analysis strategy is considered within the IMPROVE trial, where we find that the conclusion, that eEVAR is relatively cost‐effective, is robust to a wide range of departures from the assumption that QoL data are MAR. More widely, this framework can help future CEA and health economics studies communicate the impact of alternative assumptions about the missing data to decision makers and health policy makers, for whom concern about missing data may lead to the study's findings being misinterpreted or disregarded. These may include settings where a wider range of endpoints including costs, but also survival or no events are liable to be MNAR.

This paper extends standard Bayesian approaches developed for CEA that use clinical trials (Baio, 2013; Nixon & Thompson, 2005) to address the typical concern that data are likely to be MNAR in this setting. Here, a fully Bayesian approach has several advantages. First, the uncertainty about the missing values is automatically and coherently propagated through the model and reflected in the quantities calculated for decision making. Second, Bayesian models provide scope for incorporating expert information about the missingness through the priors, while accommodating typical features in CEA such as bivariate endpoints and covariate adjustment. Software code to implement these models is provided to encourage future CEA studies to perform sensitivity analysis to alternative missing data assumptions. Third, these models naturally extend to more complex settings, for example, to allow for MNAR for other endpoints (e.g., costs), to allow for clustering, or to adjust for observed confounding in CEA that use observational data.

An essential element of the proposed framework is the elicitation tool, which was specifically tailored to address key features in the CEA setting. In particular, the elicitation questionnaire had to recognise the scale and form of the QoL endpoint in question, and to recognise that the expected value and uncertainty about the missing value may differ by treatment arm. Our elicitation approach also provided the flexibility to address a major challenge in elicitation studies, which is to allow for correlation in the elicited values across the treatment arms. The elicitation tool developed as part of this research complements the tools already available for eliciting expert opinion for the parameters required in CEA. For example, the Sheffield Elicitation Framework is designed primarily for facilitating face‐to‐face meetings with groups of experts and behavioral (consensus) approaches to synthesise expert beliefs (Oakley & O'Hagan, 2010). Previous elicitation exercises in CEA using face‐to‐face meetings have resulted in relatively small sample sizes (five or fewer experts; Bojke et al., 2010; Garthwaite, Chilcott, Jenkinsdon, & Tappenden, 2008). Further, reaching a group consensus tends to impose a considerable burden on the experts and analysts, and may lead to relatively narrow uncertainty intervals (overconfident judgements; Soares et al., 2011). Our approach can be fully implemented online, and with a mathematical approach to combine individual, independently elicited beliefs. This practical approach enables the study to minimise the burden on experts, and facilitates the elicitation exercise across a reasonable number and range of experts. We recognise that in some settings, for example, technologies within the same therapeutic area, it would be unnecessary to repeat the full elicitation exercise. The questions in our elicitation exercise were framed to inform priors for sensitivity parameters about alternative missing data mechanisms, but the proposed tool has a broader application, for example, to elicit priors for other parameters as part of sensitivity analyses to address parameter or structural uncertainty in CEA that do not have access to individual patient data.

The proposed sensitivity analysis framework has some limitations. Although the Bayesian framework can accommodate different levels of complexity, the presence of missing covariates complicates the sensitivity analysis considerably. Within our motivating example, one incomplete covariate, the Hardman index, was accommodated. However, in clinical trials or indeed observational studies with many incomplete covariates, computational issues may arise, particularly if both incomplete outcomes and covariates lead to a small proportion of complete cases. Additionally, we have focused on eliciting expert opinion for a single time point. In practice, QoL is likely to be measured (and missing) at several points in time. “Longitudinal” settings raise further challenges for the elicitation because the sensitivity parameters are likely to be correlated over time, and the experts may be subject to recall biases.

This research has advanced methods for handling missing data in CEA, with relevance to health economics studies that use patient‐reported outcomes more widely, and has opened up new research directions. First, our approach jointly models the cost‐effectiveness endpoints, assuming bivariate normality. However, QoL data is typically left skewed, bimodal, and with spikes at 0 and/or 1, and in future, the proposed pattern‐mixture model can be extended to allow for these features. Second, we plan to extend our elicitation tool to allow for greater flexibility in the distribution of possible values elicited, for example, by using nonparametric approaches (Chaloner, 1996). Third, although the framework extends to a longitudinal setting, it becomes increasingly complex where there are many time‐points. The elicitation of all the required quantities to allow for different, correlated Δ over time is challenging, and may require the analyst to make some simplifying assumptions. Further, although the proposed model is very general, it does not allow the Δs to vary with dropout time. Selection models can offer an alternative approach for addressing these challenges, and work in this area is underway. Nevertheless, we have demonstrated that our proposed framework is accessible to experts, computationally feasible and hence provides a broad practical framework for inference about cost‐effectiveness when a nontrivial proportion of data are missing.

## Supporting information

MissingQoL‐HealthEconomics‐supplement.pdf

## APPENDIX A

### A.1

#### A.1.1

The specification of the joint model for the primary analysis of the data from the IMPROVE trial is implemented using WinBUGS as follows.

First, a joint distribution for the QoL scores at 3 and 12 months (QoL_1_ and QoL_2_) is specified as the product of a marginal distribution for QoL_1_ and a conditional distribution for QoL_2_ given QoL_1_. For QoL_1_

(A1) $$\begin{array}{ll} {\text{QoL}}_{i1}∼N(\mu_{i1},\sigma_{1}^{2}),\;\;\mu_{i1}\;\text{as in}\;(6). & \; \end{array}$$

The specification for QoL_2_ is

(A2) $$\begin{array}{ll} \begin{array}{ccc} {\text{QoL}}_{i2} & ∼N(\varphi_{i},\sigma_{2}^{2}(1-\varrho^{2})) & \\ \varphi_{i} & =\mu_{i2}+\frac{\varrho \sigma_{2}}{\sigma_{1}}({\text{QoL}}_{i1}-\mu_{i1}),\;\;\mu_{i2}\;\text{as in}\;(6) \end{array} & \; \end{array}$$

where *σ*_2_ and *ϱ* represent the marginal standard deviation for QoL_*i*2_, and the correlation between QoL_*i*1_ and QoL_*i*2_, respectively.

Second, the 12 month QALY is calculated from the observed/imputed QoL at 3 and 12 months assuming linear interpolation, according to the “area under the curve” method (Equation 1).

$${\text{QALY}}_{i}=\sum_{j=1}^{J}{\text{QoL}}_{ij}\times t_{ij}=\left(\frac{{\text{QoL}}_{i1}}{2}\times \frac{1}{4}\right)+\left(\frac{{\text{QoL}}_{i1}+{\text{QoL}}_{i2}}{2}\times \frac{3}{4}\right).$$

As *q* *a* *l* *y*_*i*_ is a linear combination of two dependent normal distributions, QoL_*i*1_ and QoL_*i*2_, by properties of normal distributions, it also has a normal distribution s.t.

$${\text{QALY}}_{i}=\frac{1}{2}{\text{QoL}}_{i1}+\frac{3}{8}{\text{QoL}}_{i2}∼N(\frac{1}{2}\mu_{i1}+\frac{3}{8}\mu_{i2},\frac{1}{4}\sigma_{1}^{2}+\frac{3}{8}\varrho \sigma_{1}\sigma_{2}+\frac{9}{64}\sigma_{2}^{2})=N(\nu_{i}^{q},\sigma_{q}^{2}).$$

Third, 12‐month costs (Cost) are modeled assuming bivariate normality with the 12‐month QALYs by specifying a conditional distribution of costs given QALYs:

$$\begin{array}{ccc} {\text{Cost}}_{i} & ∼N(\phi_{i},\sigma_{c}^{2}(1-\rho^{2})) & \\ \phi_{i} & =\nu_{i}^{c}+\rho \frac{\sigma_{c}}{\sigma_{q}}({\text{QALY}}_{i}-\nu_{i}^{q}),\;\;\nu_{i}^{c}\;\text{as in}\;(6). \end{array}$$

All the regression coefficients are assigned normal priors, centered on 0 with large variance, the standard deviations are given flat priors on the log scale over a range supported by the data ( $\log(\sigma_{1})∼\text{uniform}(-10,5)$, $\log(\sigma_{2})∼\text{uniform}(-10,5)$ and $\log(\sigma_{c})∼\text{uniform}(-5,50)$) and the correlations, *ϱ* and *ρ*, are given uniform( − 1,1) priors.
