# Supplementary material for: A Bayesian framework for health economic evaluation in studies with missing data
Source: Health Econ. 2018 Jul 3;27(11):1670–83. doi: 10.1002/hec.3793 (PMC6220766; doi:10.1002/hec.3793)
Supplement: Supplementary file 1 — MissingQoL‐HealthEconomics‐supplement.pdf [file HEC-27-1670-s001.pdf]

A Bayesian framework for health economic evaluation in studies with missing data
Supplementary Material

In this supplementary material we provide additional details about the model specification, the missing data in the IMPROVE trial and the full model code.

S1 Further details about the model specification

We developed a suitable model for cost-effectiveness analysis (CEA) of the IMPROVE trial allowing the missing QoL outcomes to be 'missing not at random' (MNAR), but assuming that the missing cost and Hardman index were 'missing at random' (MAR). For ease of modelling, we categorised the patients into 4 types: A) eligible patients who survived to 12 months, B) eligible patients who died between 3 and 12 months, C) eligible patients who died before 3 months and D) patients who were ineligible to follow-up. Using a Bayesian framework we built an appropriate model from a series of sub-models, allowing different levels of complexity for the 4 categories of patients. We jointly modelled the two endpoints as described in Section 3, and extended the model to incorporate a covariate imputation model (for the Hardman index), and to calculate the cost-effectiveness estimates of interest.

An overview of how these sub-models fit together is provided by Figure S1. The sub-models for the Category A and B patients are built from multiple components: a pattern-mixture model (PMM) for the EQ-5D scores, calculation of QALYs using observed and imputed EQ-5D scores and a cost model conditional on QALYs. The Hardman index is imputed as a categorical variable taking account of age and sex, and feeds into the sub-models for the patients in Categories A and B. The calculation of the CEA outputs draws

from the other sub-models to calculate differences in overall mean QALYs and costs, and finally incremental net benefits (INB).

Figure S1: Overview of model structure

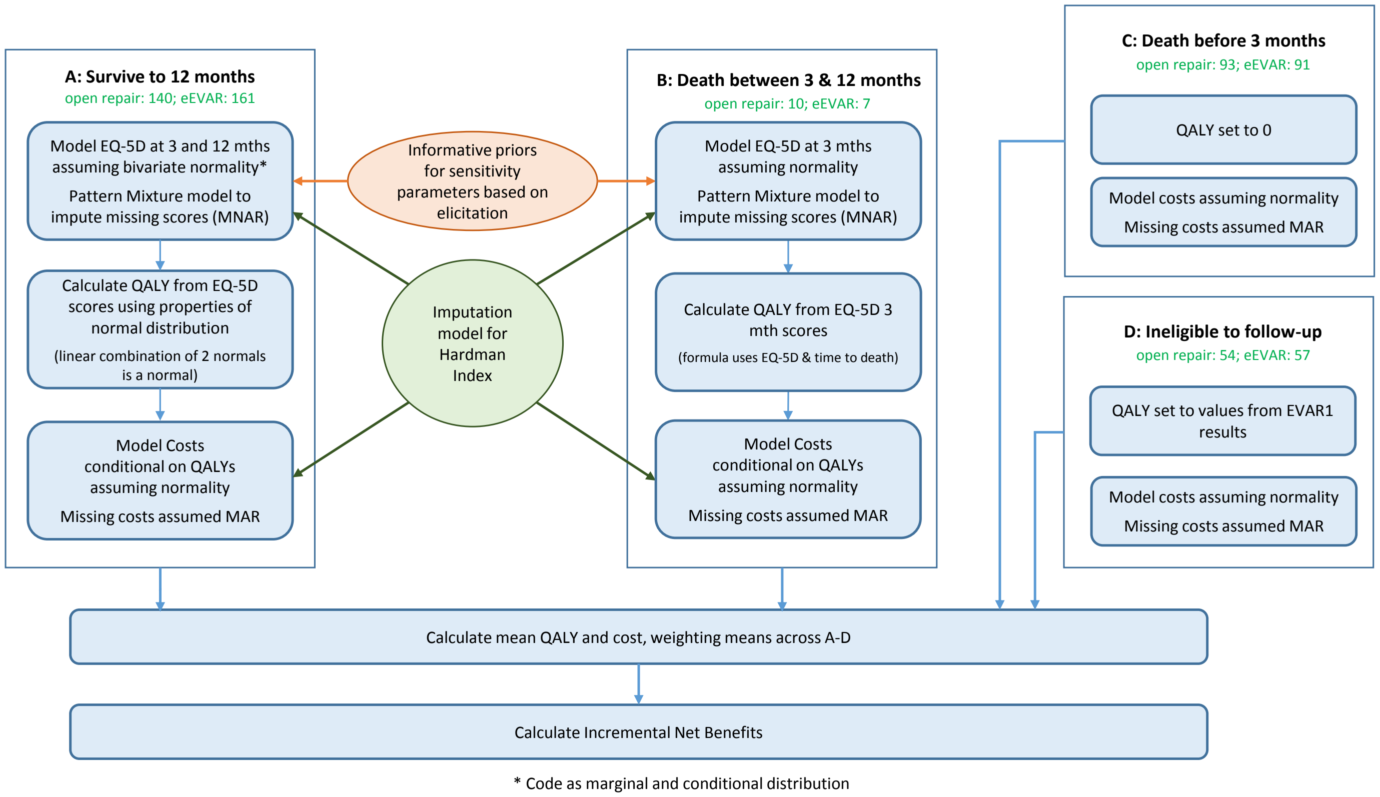

The number of patients in each category are shown in green by treatment arm.

The sub-model for the 12 month survivors who were eligible to follow-up (Category A patients) was described in detail in Section 3, so here we restrict our discussion to the other parts of the model. The sub-model for the patients who died between 3 and 12 months (Category B) consists of 3 components, analogous to those specified for the Category A sub-model. However, the first component simplifies as the QALY calculation for these patients depends solely on the EQ-5D at 3 months, so only a pattern mixture model for QoL_1 is required (defined as for Category A). The QALY calculation now uses QoL_1 and time to death in days (ttd):

$$\text{QALY}_i = \frac{1}{2}\text{QoL}_{i1} \times \frac{ttd_i}{365} \sim N\left(\frac{ttd}{2 \times 365}\mu_{i1}, \left(\frac{ttd}{2 \times 365}\right)^2 \sigma_1^2\right). \tag{S1}$$

The sub-models for the 2 remaining categories are simplified further. Following the original CEA we make assumptions about the QALY values, and so there is no need to model QoL at either time-point and the costs models are set up without conditioning on the QALYs and covariate adjustment. Patients who die before 3 months in both categories are assumed to have a QALY of 0. QALYs are calculated for the remaining Category D patients assuming that their QoL values are the same as for a previous trial that compared these interventions for elective patients (EVAR1), i.e. 0.75 and 0.74 at 3 and 12 months respectively (Brown *et al.*, 2012).

References

Brown, L. C., Powell, J. T., Thompson, S. G., Epstein, D. M., Sculpher, M. J., and Greenhalgh, R. M. (2012). The UK EndoVascular Aneurysm Repair (EVAR) trials: randomised trials of EVAR versus standard therapy. *Health Technology Assessment*, **16**, (9), 1–218.

S2 Further details about the missing IMPROVE data

Table S1 shows the patterns of the missing data for survivors at 12 months.

Table S1: Pattern of missing data for patients who survived for 12 months (Category A)

missing variables⋆	number of records†	number of missing variables‡
none	138	0
eq3	3	1
eq12	4	1
cost	44	1
hard	12	1
eq3 and cost	18	2
eq12 and cost	27	2
eq3 and hard	2	2
eq12 and hard	1	2
cost and hard	9	2
eq3, eq12 and cost	31	3
eq3, cost and hard	3	3
eq12, cost and hard	6	3
eq3, eq12, cost and hard	3	4
total	301	309

⋆ eq3 = EQ-5D at 3 months; eq12 = EQ-5D at 12 months; hard = Hardman index
† number of records for the missingness pattern
‡ number of missing variables in the missingness pattern

S3 BUGS code

```
# WinBUGS model code for Bayesian CEA of IMPROVE trial data
# Missing data assumptions:
#    EQ-5D (outcome) - MNAR; Costs (outcome) - MAR; Hardman index (covariate) - MAR
# Variables:
#    EQ[1:613,1:2] - EQ-5D scores for 613 individuals at 3 and 12 months
#    cost[1:613] - costs for 613 individuals
#    cage[1:613] - centered age
#    sex[1:613] - binary indicator of gender: male=0, female=1
#    hard[1:613] - Hardman index: categories 1-5

model{
   ##### Sub-model for Category A patients
   # -------------------------------------------------------------------------------------
   for(i in 1:N[1]) { # loop through individuals who survived to 12 months
      # marginal distribution for EQ-5D at 3 months
      EQ[i,1] ~ dnorm(mu[i,1],tau.e3[1])
      mu[i,1] <- eta[group[i],1] + beta.age[1]*cage[i] + beta.sex[1]*sex[i] + beta.hard[1,hard[i]]
         + delta[group[i]]*miss[i,1] + (eta[1,1]-eta[2,1])*miss[i,1]*(group[i]-1)
      # conditional distribution of EQ-5D at 12 months given EQ-5D at 3 months
      EQ[i,2] ~ dnorm(varphi[i],inv.chi2)
      varphi[i] <- mu[i,2] + alpha*(EQ[i,1] - mu[i,1])
      mu[i,2] <- eta[group[i],2] + beta.age[2]*cage[i] + beta.sex[2]*sex[i] + beta.hard[2,hard[i]]
         + delta[group[i]]*miss[i,2] + (eta[1,2]-eta[2,2] )*miss[i,2]*(group[i]-1)
      # calculate QALY from EQ-5D scores
      QALY[i] <- (EQ[i,1]/2) + (EQ[i,2]*3/8)
      Qmu[i] <- (mu[i,1]/2) + (mu[i,2]*3/8)
      # conditional distribution of costs given QALYs
      cost[i] ~ dnorm(phi[i],inv.psi2[1])
      phi[i] <- Cmu[group[i],1] + beta.age[3]*cage[i] + beta.sex[3]*sex[i] + beta.hard[3,hard[i]]
         + beta*(QALY[i]-Qmu[i])
   }
   alpha <- varrho * sigma.e12/sigma.e3[1]
   beta <- rho[1] * sigma.c[1]/sigma.q
   # marginal variance and sd for EQ-5D at 12 months
   sigma2.e12 <- pow(sigma.e12,2)
   sigma.e12 <- exp(logsigma.e12)
   # conditional variance and precision for EQ-5D at 12 months
   chi2 <- sigma2.e12 * (1 - pow(varrho,2))
   inv.chi2 <- 1/chi2
   # variance and sd for QALY
   sigma2.q <- (sigma2.e3[1]/4) + (varrho * sigma.e3[1] * sigma.e12  * 3/8) + (sigma2.e12 * 9/64)
   sigma.q <- sqrt(sigma2.q)
   # -------------------------------------------------------------------------------------

   ##### Sub-model for Category B patients
   # -------------------------------------------------------------------------------------
   for (i in N[1]+1:N[2]) { # loop through individuals who died between 3 and 12 months
      # distribution for EQ-5D at 3 months
      EQ[i,1] ~ dnorm(mu[i,1],tau.e3[2])
      mu[i,1] <- zeta[group[i]] + beta.age[1]*cage[i] + beta.sex[1]*sex[i] + beta.hard[1,hard[i]]
        + delta[group[i]]*miss[i,1] + (zeta[1]-zeta[2])*miss[i,1]*(group[i]-1)
      # calculate QALY from EQ-5D scores
      const[i] <- tdeath[i]/(2*365)
```

```
   QALY[i] <- EQ[i,1] * const[i]
   Qmu[i] <- mu[i,1] * const[i]
   sigmaB2.q[i] <- sigma2.e3[2]*pow(const[i],2)
   sigmaB.q[i] <- sqrt(sigmaB2.q[i])
   gamma[i] <- rho[2] * sigma.c[2]/sigmaB.q[i]
   # conditional distribution of costs given QALYs
   cost[i] ~ dnorm(phi[i],inv.psi2[2])
   phi[i] <- Cmu[group[i],2] + gamma[i]*(QALY[i]-Qmu[i])
}
# ------------------------------------------------------------------------------------------

### combined code for sub-models for Category A and B patients
# ------------------------------------------------------------------------------------------
# marginal precision, variance and sd for EQ3
for (k in 1:2) { # loop through patient categories A-B
   tau.e3[k] <- 1/sigma2.e3[k]
   sigma2.e3[k] <- pow(sigma.e3[k],2)
   sigma.e3[k] <- exp(logsigma.e3[k])
}
# conditional variance and precision for cost
for (k in 1:2) { # loop through patient categories A-B
   psi2[k] <- sigma2.c[k] * (1 - pow(rho[k],2))
   inv.psi2[k] <- 1/psi2[k]
}
# ------------------------------------------------------------------------------------------

##### Sub-model for Category C patients
# ------------------------------------------------------------------------------------------
for (i in N[2]+1:N[3]) { # loop through individuals who died before 3 months
   cost[i] ~ dnorm(Cmu[group[i],3],tau.c[3]) # distribution for costs
}
# ------------------------------------------------------------------------------------------

##### Sub-model for Category D patients
# ------------------------------------------------------------------------------------------
for (i in N[3]+1:N[4]) { # loop through individuals who are ineligible to follow-up
   cost[i] ~ dnorm(Cmu[group[i],4],tau.c[4]) # distribution for costs
}
# ------------------------------------------------------------------------------------------

### combined code for sub-models for Category A to D patients
# ------------------------------------------------------------------------------------------
# marginal variance and sd for cost
for (k in 1:4) { # loop through patient categories A-D
   tau.c[k] <- 1/sigma2.c[k]
   sigma2.c[k] <- pow(sigma.c[k],2)
   sigma.c[k] <- exp(logsigma.c[k])
}

# priors
for(g in 1:2) { # loop through treatment arms
   for (k in 1:4) { # loop through patient categories A-D
      Cmu[g,k] ~ dnorm(0,1.0E-10)
   }
   for(t in 1:2) { # loop through time-points: 3 and 12 months
      eta[g,t] ~ dnorm(0,1.0E-6)
```

```
   }
   zeta[g] ~ dnorm(0,1.0E-6)
}
for (k in 1:2) {logsigma.e3[k] ~ dunif(-10,5)} # loop through patient categories A-B
logsigma.e12 ~ dunif(-10,5)
for (k in 1:4) {logsigma.c[k] ~ dunif(-5,50)} # loop through patient categories A-D
varrho ~ dunif(-1,1)
for (k in 1:2) {rho[k] ~ dunif(-1,1)} # loop through patient categories A-B

# informative prior for sensitivity parameters: a mixture of multivariate normals
delta[1:2] ~  dmnorm(delta.mu[1:2,pick],delta.Omega[1:2,1:2,pick])
pick ~ dcat(Q[])
for (i in 1:Nexpert) {Q[i] <- 1/Nexpert} # loop through experts

# priors on covariates
for (k in 1:3) { # loop through EQ-5D at 3 months, EQ-5D at 12 months and costs
   beta.age[k] ~ dnorm(0,1.0E-6)
   beta.sex[k] ~ dnorm(0,1.0E-6)
   beta.hard[k,1] <- 0 # alias first level of hardman index beta
   for (j in 2:4) {beta.hard[k,j] ~ dnorm(0,1.0E-6)}
   beta.hard[k,5] <- beta.hard[k,4]
   beta.hard[k,6] <- beta.hard[k,4]
}
# ------------------------------------------------------------------------------------------

##### Sub-model for covariate imputation for Hardman index
# ------------------------------------------------------------------------------------------
for (i in 1:N[4]){ # loop through all patients
   hard[i] ~ dcat(P[i,1:6])
   for (g in 1:6) { # loop through Hardman index categories
      P[i,g] <- iota[i,g]/sum(iota[i,])
      log(iota[i,g]) <- kappa0[g] + kappa.age[g] * cage[i] +  kappa.sex[g] * sex[i]
   }
}
kappa0[1] <- 0 # constraint iota[i,1]=1
kappa.age[1] <- 0 # constraint iota[i,1]=1
kappa.sex[1] <- 0 # constraint iota[i,1]=1
for (g in 2:6) { # loop through Hardman index categories, excluding first category
   kappa0[g] ~ dnorm(0,1.0E-6)
   kappa.age[g] ~ dnorm(0,1.0E-6)
   kappa.sex[g] ~ dnorm(0,1.0E-6)
}
# ------------------------------------------------------------------------------------------

##### Sub-model for calculation of CEA outputs
# ------------------------------------------------------------------------------------------
# calculate effectiveness measures

# individuals who survived to 12 months (Category A patients)
# calculate mean QALY for OPEN arm
Qave[1,1] <- ((eta[1,1]+delta[1] * pc.mis[1,1])/2) + ((eta[1,2]+delta[1] * pc.mis[1,2])*3/8)
# calculate mean QALY for EVAR arm (sensitivity parameter is difference from observed OPEN mean)
mth3.contribution <- (eta[2,1] * (1-pc.mis[2,1]) + (eta[1,1]+delta[2]) * pc.mis[2,1])/2
mth12.contribution <- (eta[2,2] * (1-pc.mis[2,2]) + (eta[1,2]+delta[2]) * pc.mis[2,2])*3/8
Qave[2,1] <- mth3.contribution + mth12.contribution
```

```
    # individuals who died between 3 and 12 months (Category B patients)
    for (i in N[1]+1:N[2]) {
       open.flag[i] <- 2-group[i]
       evar.flag[i] <- group[i]-1
    }
    # OPEN arm
    Qave[1,2] <- inprod(Qmu[N[1]+1:N[2]],open.flag[N[1]+1:N[2]])/sum(open.flag[N[1]+1:N[2]])
    # EVAR arm
    Qave[2,2] <- inprod(Qmu[N[1]+1:N[2]],evar.flag[N[1]+1:N[2]])/sum(evar.flag[N[1]+1:N[2]])

    # individuals who are ineligible to follow-up (Category D patients)
    Qave[1,4] <- 0.3087 # OPEN arm
    Qave[2,4] <- 0.2951 # EVAR arm

    # calculate overall mean QALYs and costs, weighting means across Categories A-D
    for (g in 1:2) { # treatment arms
       Qave[g,3] <- 0 # death before 3 months
       Qaverage[g] <- inprod(Qave[g,],pc.type[g,])
       Caverage[g] <- inprod(Cmu[g,],pc.type[g,])
    }

    incQ <- Qaverage[2] - Qaverage[1] # increment in QALYs
    incC <- Caverage[2] - Caverage[1] # increment in costs

    # calculate incremental net benefits (INB)
    for (j in 1:M) { # loop through values of efficacy (QALY) gains
       inb[j] <- (threshold[j]*incQ)-incC
       p.ce[j] <- step(inb[j]) # probability INB are positive
    }
    # ---------------------------------------------------------------------------------------
}
```
